# Comparative Analysis of Epidemiological Outcome of Incidence, Mortality and Lethality by COVID-19 between the States of Espírito Santo and Minas Gerais, Brazil

**DOI:** 10.3390/epidemiologia5020017

**Published:** 2024-06-02

**Authors:** Leonardo Gomes da Silva, Italla Maria Pinheiro Bezerra, Gabriella Lima Santos, Luiz Carlos de Abreu

**Affiliations:** 1 Study Design and Scientific Writing Laboratory, Federal University of Espírito Santo (UFES), Vitória 29043-900, Brazil; gabriellalima@outlook.com (G.L.S.); luiz.abreu@ufes.br (L.C.d.A.); 2 Postgraduate Program in Public Health, Health Sciences Center, Federal University of Espírito Santo (UFES), Vitória 29043-900, Brazil; 3 Department of Nursing, School of Sciences of Santa Casa de Misericórdia de Vitória (EMESCAM), Vitória 29045-402, Brazil; 4 Postgraduate Program in Public Policies and Local Development, School of Sciences of Santa Casa de Misericórdia de Vitória (EMESCAM), Vitória 29045-402, Brazil; italla.bezerra@emescam.br; 5 Associate Clinical Professor at University of Limerick, V94 T9PX Limerick, Ireland

**Keywords:** coronavirus, COVID-19, mortality, incidence, lethality

## Abstract

At the beginning of December 2019, a new type of coronavirus emerged, SARS-CoV-2. This virus causes COVID-19, a highly contagious disease that can initially present asymptomatically and can also lead to death. Our ecological study goal was to evaluate the incidence, mortality, and lethality rates for COVID-19 between the states of Espírito Santo and Minas Gerais, with time series analysis using secondary and public databases on COVID-19 from January 2020 to December 2022. Prais–Winsten linear regression was used for trend analyses. In 2020, the rate in Espírito Santo was 2.19 times greater than in Minas Gerais. This trend continued in 2021, with Espírito Santo’s rate being 1.29 times greater. In 2022, Espírito Santo’s rate remained 2.65 times higher than Minas Gerais. Furthermore, Espírito Santo had the highest mortality, with the exception of 2021. In turn, Minas Gerais had the highest fatality rate throughout the analyzed pandemic period. The state of Espírito Santo had a higher incidence of COVID-19, as well as higher mortality when compared to the state of Minas Gerais. Furthermore, both states showed similar trends for mortality, lethality, and incidence in the years 2020 and 2021.

## 1. Introduction

The narrative commences in December 2019, precisely within the confines of Wuhan, China, where a novel strain of coronavirus, SARS-CoV-2, was first detected and efforts have been made to counteract the virus responsible for the pandemic. It is worth noting that this newfound strain of coronavirus has given rise to the emergence of COVID-19, a swiftly spreading ailment with transmission modes that are challenging to manage [1].

Regarding the impacts caused in the economic sphere, jurisdictional tax measures and exemptions were adopted in response to combating COVID-19. Such measures were taken in order to encourage acceptance by the population. Regarding social impacts, it also reveals that there were changes in lifestyle, limitations on travel, and bans on meetings, among others [2].

Currently, data provided through the World Health Organization (WHO) Coronavirus (COVID-19) Dashboard shows that by the month of August 2023, globally, 770,085,713 confirmed cases of COVID-19 infection have been recorded. In addition, 6,956,173 deaths have been confirmed due to the disease. Among these records, Europe is the continent with the highest cumulative number of confirmed cases [3].

According to data highlighted in the weekly epidemiological update on COVID-19 made available by the World Health Organization (2023) [4], there was a global increase in the number of new cases and deaths due to COVID-19 until mid-January 2023, which is why they included it in the new document updates on the variants and subvariants of concern, as well as records on the increase in the number of hospitalizations in several countries, including in the Intensive Care Unit (ICU).

However, in the last update of the global document made available by the WHO (2023) [5], which concerns the month of August 2023, there is information on the decrease in the number of deaths (50%), despite the increase (38%) in the number of confirmed cases, in addition to the decrease in the number of ICU admissions.

Having been considered a new disease, the virus in question behaves similarly to viruses that cause pandemics, which results in similar adoption of measures to contain transmission. This transmission is classified as community transmission, given that SARS-CoV-2 remains stable on surfaces such as plastic and stainless steel for up to three days, in addition to being transmissible in aerosol form for hours when outside the human body [6]. 

In this aspect, a study has indicated a potential relationship between the incidence of COVID-19 and temperature, considering that temperature affects human behavior and interaction with the environment, thereby influencing the dynamics of disease transmission. Additionally, it has been demonstrated that between 17 °C and 24 °C, the transmission of COVID-19 is reduced, as it directly impacts the potential for virus aerosolization through particles in the environment [7]. 

Furthermore, Li et al. [8] describe that infection with SARS-CoV-2 can initially be asymptomatic and can progress seriously and even lead to death, and can affect all ages. In this sense, Zhang et al. [9] point out that SARS-CoV-2 variants represent a great threat to the prevention and control of COVID-19 transmission, given the ability of new strains to be imported between different countries.

In this context, Abate et al. [10] point out in research on admissions to Intensive Care Units that the mortality rate reached 39% of patients infected with the new coronavirus admitted to this sector. Furthermore, it points out that around 66% of patients admitted to the ICU had associated comorbidities, which in turn, through regression analysis, detected that the comorbidity increased the risk of death by 39%.

However, in May 2023, the end of the Public Health Emergency of International Importance regarding COVID-19 was declared, following statements about the decreasing trend in deaths due to COVID-19, a decrease in hospitalizations and ICU admissions as well as an increase in the population’s level of immunity to SARS-CoV-2 [11].

Despite the declaration, in terms of comparison between countries, Brazil is currently in 6th position in the ranking of cumulative cases, totaling 37,717,062 confirmed cases, behind countries such as the United States of America, China, India, France, and Germany [3]. In turn, considering the southeastern region of Brazil, more specifically the states Espírito Santo (ES) and Minas Gerais (MG), 1,339,629 confirmed cases and 15,110 deaths from COVID-19 were registered in ES, while MG recorded 4,209,719 cases and 65,740 deaths, until the beginning of September 2023, as published by the coronavirus panel [12].

Given this scenario, the question arises: what are the incidence, mortality, and lethality rates for COVID-19 in the states of Espírito Santo and Minas Gerais between 2020 and 2022?

The study is justified by the emerging need to carry out research in order to understand the profile of infected people in terms of incidence, mortality, and lethality rates due to infection by the new coronavirus in the states of Espírito Santo and Minas Gerais. In this way, deepening this theme may contribute to better coping with the disease by policymakers, through robust and specific evidence, in order to contribute to the development and implementation of local interventions, thus making coping with the pandemic in the states more efficient.

Therefore, the objective of the present study is to evaluate the incidence, mortality, and lethality rates for COVID-19 between the states of Espírito Santo and Minas Gerais, Brazil, from 2020 to 2022.

## 2. Materials and Methods

### 2.1. Study Design

This is a quantitative ecological study where time series analyses were conducted using a public database on COVID-19 provided by the Brazilian Ministry of Health through the website https://covid.saude.gov.br/ (accessed on 8 February 2023).

Time series analyses help organize quantitative information over time, allowing for the prediction of potential future scenarios of the disease within the population [13,14].

### 2.2. Location and Period of Research

**Figure 1:**
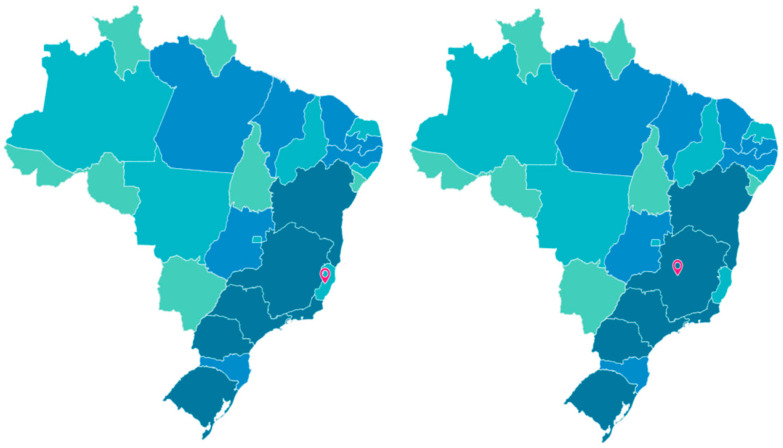
Map of Brazil with representation of the states Espírito Santo (**left**) and Minas Gerais (**right**). Source: IBGE (2023).

The states Espírito Santo and Minas Gerais were selected to carry out this research, considering that both are part of the Southeast region of Brazil (Figure 1) and border each other, where Espírito Santo has 23 cities bordering Minas Gerais [15,16,17]. 

**Figure 2:**
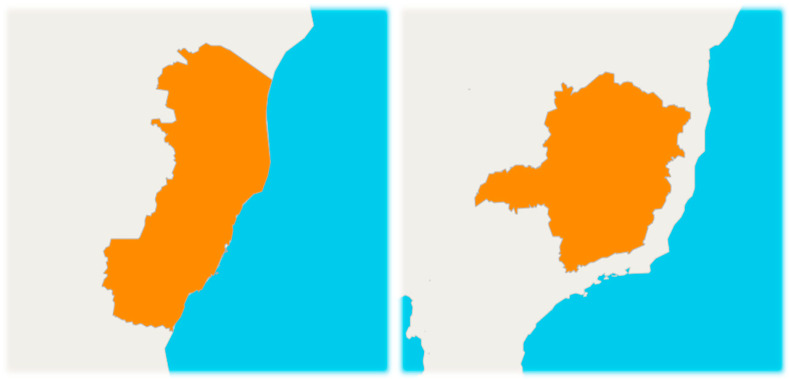
Map of the states of Espírito Santo (**left**) and Minas Gerais (**right**). Source: IBGE (2023).

According to the last census carried out by the Brazilian Institute of Geography and Statistics (IBGE) [15] in the year 2022, the state of Espírito Santo (Figure 2) had a total population of 3,833,486 people. The state has a total territorial area of 46,074.448 km^2^, with a Human Development Index (HDI) in 2021 of 0.771, occupying 5th position in the national ranking and per capita income of R$1723.

In turn, the state of Minas Gerais (Figure 2), in 2022, presented in the census the equivalent of 20,538,718 people, in a total territorial area of 586,513,983 km^2^. The state presented a Human Development Index (HDI) in 2021 of 0.774, occupying 4th position in the national ranking and nominal monthly income per capita of R$1529 [16].

Comparing the two states, it is noticeable that the state of Minas Gerais is approximately 12 times larger than the state of Espírito Santo, as well as having a larger population, roughly five times, and a higher HDI as well.

The data collected pertained to the period spanning from January 2020 to December 2022.

### 2.3. Study Population and Eligibility Criteria

The population of the states of Espírito Santo and Minas Gerais was used, and all confirmed cases and deaths related to COVID-19 were included in the study using the International Classification of Diseases, 10th edition (ICD-10), of “B34.2, Coronavirus infection of unspecified location” and “U07.1 COVID-19, identified virus” or “U07.2 COVID-19, unidentified virus”. It should be noted that all cases and deaths included in the research are confirmed.

Data that did not provide complete information regarding the state were excluded from collection, making identification unfeasible.

### 2.4. Data Collection

The data on confirmed cases and confirmed deaths were extracted from the Coronavirus Panel, available on the website https://covid.saude.gov.br/, on 8 February 2023. This panel was created by the Brazilian Ministry of Health and designed to be the official and public vehicle for communication about the epidemiological situation of COVID-19 in the country, registered by the State and Municipal Health Secretariats.

Data on the resident population of the states of Espírito Santo and Minas Gerais were extracted from the projection base of the population of Brazil of the Federation Units by sex and year for the period 2000–2030 from the Brazilian Institute of Geography and Statistics (IBGE) [15,16] available in the database of the Department of Informatics of the Unified Health System (DATASUS) [18], on the website https://datasus.saude.gov.br/informacoes-de-saude-tabnet/ (accessed on 8 February 2023).

DATASUS provides information that can serve to support objective analyses of the health situation, evidence-based decision making, and the development of health action programs. It is important to highlight that data collection was conducted by two independent researchers, and the data were organized into Microsoft Excel spreadsheets. A comparison of the collected data and totals of each variable was performed to mitigate collection bias. 

### 2.5. Study Variables

Dependent (outcome): confirmed cases, confirmed deaths, fatality rate, mortality and incidence.

Independent (explains the outcome): years 2020, 2021 and 2022.

### 2.6. Data Analysis

The data were tabulated in a Microsoft Excel 365 spreadsheet, version 2019, to ensure the organization of absolute frequencies, taken from the public platform, by variable, with the independent and dependent variables. Thus, the relative frequency was calculated, obtaining data on the number of cases and deaths confirmed by COVID-19 in the month and/or year and divided by the total number of the period evaluated and multiplied by 100, expressed as a percentage (%).

The incidence rate relies crucially on the recording of new cases of a disease, serving as a measure of events and can be used as an estimate of the risk of falling ill, as it uses in its denominator the population at risk of developing the disease. Meanwhile, the mortality rate serves as a measure of disease severity and the risk of death from the disease, as its denominator includes the entire population, both sick and not sick. Conversely, the case fatality rate is not a rate but is expressed as a percentage and its denominator is limited to the sick population. Therefore, the incidence and mortality rates were expressed per 100,000 inhabitants, and lethality was expressed as a percentage (%), following the formulas, respectively:

$$Incidence=\frac{number\;of\;confirmed\;cases}{population}\times 100,000\;inhabitants$$

$$Mortality=\frac{number\;of\;confirmed\;deaths}{population}\times 100,000\;inhabitants$$

$$Lethality=\frac{number\;of\;confirmed\;deaths}{number\;of\;confirmed\;cases}\times 100\%$$

For trend analyses, the methods proposed by Antunes and Cardoso [13] were used, where time series construction rates were calculated using the Prais–Winsten regression model, which allowed first-order autocorrelation corrections to be performed. in values, organized by time. Thus, the following values were estimated: angular coefficient (β) and respective probability (p), considering a significance level of 95% confidence interval (95% CI).

The data modeling process included transformation rates (dependent variable = Y value) into a base 10 logarithmic function. The independent variable (X value) was the days or months of the historical series. The Durbin–Watson test was also used to measure the existence of first-order autocorrelation of the time series composed of daily coefficients and to verify whether the correlation was compatible with the hypothesis of random distribution regression residuals.

The results of the logarithmic rates (β) of the Prais–Winsten regression made it possible to estimate the percentage variation in daily change (Daily Percent Change—DPC) in each state, with the respective confidence intervals (95% CI), expressed by the following formulas:

DPC=(10^β−1)×100%

IC95%=[−1+10^βmin]×100%;[−1+10^βmax]×100%

In this way, it was possible to determine the rates as increasing, decreasing, or stationary and quantify the percentage variation in daily incidence, mortality, and lethality rates. The trend was considered stationary when the coefficient was not significantly different from zero (*p* > 0.05). When the coefficient value was statistically significant (*p* < 0.05), the trends were classified as increasing or decreasing according to the β value. To facilitate visualization of lethality trends, random variation in the graph was reduced using the five-order moving average technique. All statistical analyses were performed using STATA 17.0 statistical software [19].

### 2.7. Legal and Ethical Aspects

By using only aggregated secondary data from the public domain, the work meets the ethical considerations set out in Resolution of the National Health Council (CNS) No. 466, of 12 December 2012 and in Resolution 510, of 7 April 2016, and is exempt from evaluation by the Research Ethics Committee and the National Commission for Ethics in Research on Human Beings.

## 3. Results

In the state of Espírito Santo, from January 2020 to December 2022, a total of 1,313,710 cases and 15,400 deaths confirmed by COVID-19 were recorded. Table 1 shows the monthly distribution of cases and deaths confirmed by COVID-19 in Espírito Santo over time (2020 to 2022).

In the state, the first confirmed case of COVID-19 was registered in February 2020, corresponding to 0.0001% of the total cases throughout the analyzed period. As for deaths, the first records appeared in April of the same year, corresponding to a relative frequency of 0.75% of total deaths.

In 2020, in Espírito Santo, 268,768 cases and 5371 confirmed deaths from COVID-19 were recorded, with a monthly average of 22,397 and 448 respectively. The months with the highest number of confirmed COVID-19 cases were July (2.84%), November (3.53%) and December (4.16%). In relation to deaths confirmed by COVID-19, the months that stood out were June (6.88%), July (5.87%) and December (5.59%).

Continuing the pandemic period, in 2021, 371,295 cases and 8257 confirmed deaths from COVID-19 were recorded, with an average of 30,941 and 688, respectively. Here, the months of January, March, and April stand out for confirmed cases, being 3.42%, 5.02%, and 3.84%, respectively, in relation to the total period. However, for deaths, the months with the highest number of records were March (8.27%), April (13.71%), and May (8.27%).

In turn, in 2022, 673,647 cases and 1772 deaths from COVID-19 were recorded, with an average number of cases and deaths of 56,137 and 148, respectively. The month of January stands out, corresponding to 19.46% of the total cases, followed by the month of February, with 10.48% of cases, and July, with 5.87%. For all the months mentioned above, the relative frequencies of deaths also behaved as the highest, equivalent to 1.94%, 4.02%, and 1.50% respectively. It was noted that, in the annual comparison for the state of Espírito Santo, in 2022 there was a greater number of case records, with a lower number of deaths.

In turn, in the state of Minas Gerais, between January 2020 and December 2022, a total of 3,430,099 cases and 64,498 deaths confirmed by COVID-19 were recorded. In the state, the first confirmed cases of COVID-19 were registered in January 2020, corresponding to 0.004% of the total cases throughout the analyzed period and one death, in the same month.

The monthly distribution of cases and deaths confirmed by COVID-19 in Minas Gerais can be seen in Table 2.

In 2020, in the state of Minas Gerais, the average number of cases and deaths confirmed by COVID-19 was 52,890 and 1111, totaling 634,681 and 13,336, respectively. The months with the highest number of confirmed COVID-19 cases were July (2.87%), August (2.54%), and December (4.48%). In relation to deaths confirmed by COVID-19, the months mentioned above were also highlighted, corresponding in sequence to 3.76%, 3.77%, and 4.07%.

In 2021, the average number of cases was 124,184 and the number of deaths confirmed by COVID-19 was 3658. Here, the months of March, May, and June stand out for confirmed cases, being 7.77%, 5.98%, and 5.75%, respectively, in relation to the total period. However, for deaths, the months with the highest number of records were March (13.15%), April (14.36%) and May (9.64%). That year, 1,490,204 cases and 43,894 deaths were recorded. It is noteworthy that this year stands out among those analyzed for presenting the highest number of cases, as well as deaths.

In 2022, the total number of cases was 1,305,214, with a monthly average of 108,768, with the months of January (16.57%), February (8.83%) and June (5.33) standing out. As for death records, there is a total of 7268, with a monthly average of 606, highlighting the months of January, February, and July, with rates of 2.09%, 3.53%, and 1.29%, respectively.

**Figure 3:**
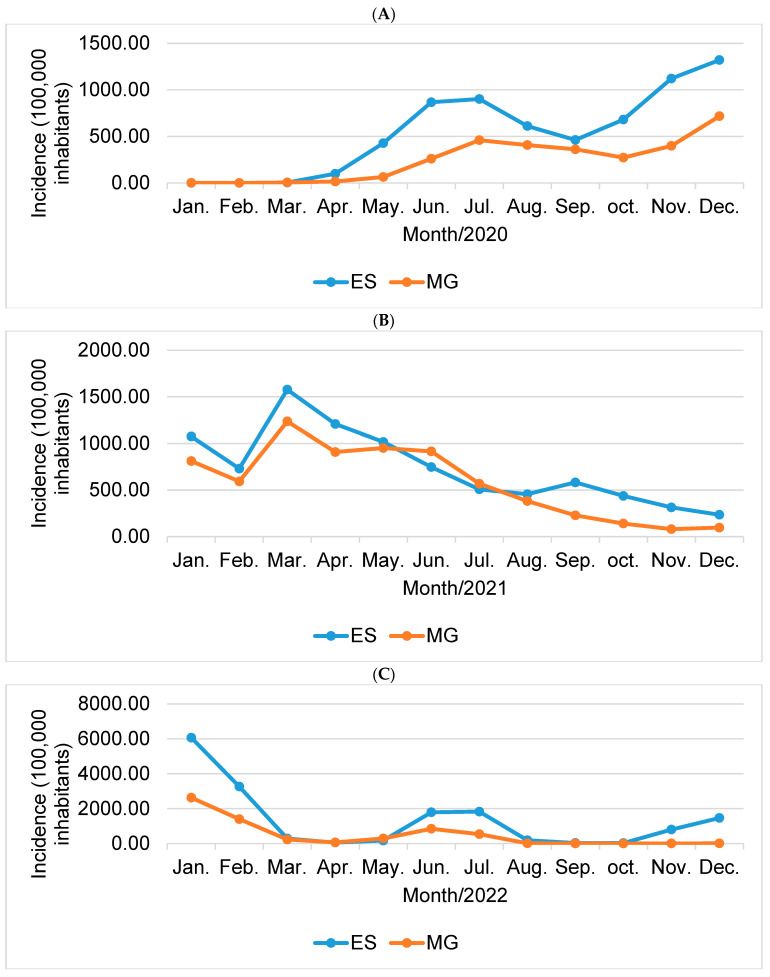
Comparative incidence graph (100,000 inhabitants) for the states Espírito Santo (ES) and Minas Gerais (MG) for the years 2020 (**A**), 2021 (**B**), and 2022 (**C**). Source: Prepared by the author (2023).

When comparing the incidences of COVID-19 between states, Espírito Santo (ES) stands out compared to the state of Minas Gerais (MG), presenting the highest rates, as in 2020 (Figure 3A), where the rate was higher in 2, 19 times and in 2021 (Figure 3B), in which the rate was 1.29 times higher than the other state. In the year 2022 (Figure 3C), the highest incidence rate for COVID-19 continued to be in Espírito Santo, with a total of 15,979.12/100,000 inhabitants, compared to a rate of 6029.64/100,000 inhabitants in the state of Minas General, which corresponds to 2.65 times higher.

To better visualize the data, see Figure 3 below, with comparative graphs between the states, year by year.

**Figure 4:**
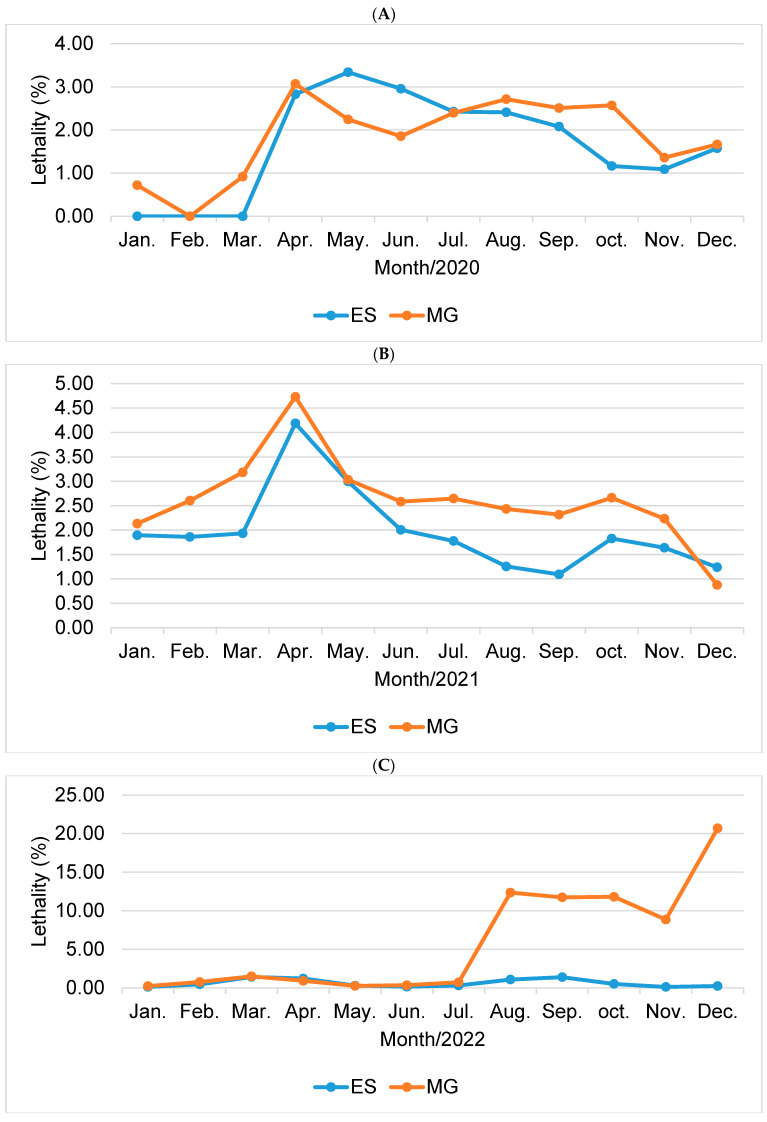
Comparative graph of lethality (%) for the states of Espírito Santo (ES) and Minas Gerais (MG) for the years 2020 (**A**), 2021 (**B**), and 2022 (**C**). Source: prepared by the author (2023).

Now, when comparing the lethality rates between the states of Minas Gerais and Espírito Santo, it was noted that during the period analyzed, the total rate remained higher in the state of Minas Gerais, with the months of April/2020 being highlighted (Figure 4A) with 3.07, April/2021 (Figure 4B) with 4.73 and December/2022 (Figure 4C) with 20.69.

Below is Figure 4, which concerns the comparison of lethality between states.

**Figure 5:**
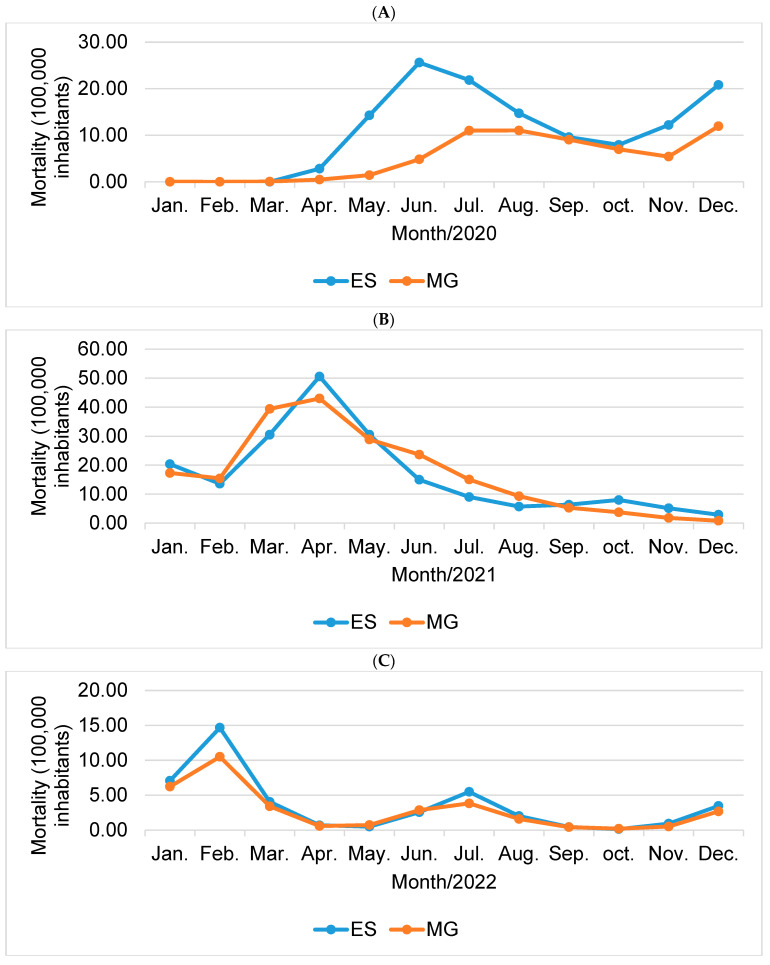
Comparative mortality graph (100,000 inhabitants) for the states Espírito Santo (ES) and Minas Gerais (MG) for the years 2020 (**A**), 2021 (**B**), and 2022 (**C**). Source: prepared by the author (2023).

Although lethality in Minas Gerais was higher throughout the analyzed period, mortality was higher than in the state of Espírito Santo only in the year 2021 (Figure 5B), reaching a rate of 203.67/100,000 inhabitants. For the years 2020 (Figure 5A) and 2022 (Figure 5C), the state of Espírito Santo recorded a mortality rate of 2.09 and 1.25 times higher than the state of Minas Gerais, respectively, as shown in Figure 5.

In addition, an analysis of trends in mortality, lethality, and incidence rates of COVID-19 in the states of Espírito Santo and Minas Gerais was carried out, which can be seen in Table 3.

After conducting the analyses, the variables considered statistically significant based on the *p*-value (*p* < 0.05) were subjected to the calculation of the daily percent change (DPC). This calculation reveals the percentage of daily change, indicating whether the variable increased or decreased during the period.

Thus, it was observed that for the years 2020 and 2021, the trends in both states were the same for mortality, lethality, and incidence. However, in 2022, there will be different behaviors between states for each variable analyzed. In the state of Espírito Santo, that year, for the variables mortality, lethality, and incidence, there was a stationary trend. In turn, Minas Gerais presents decreasing mortality (−0.62%/day), increasing lethality (+1.39%/day), and decreasing incidence (−1.80%/day).

## 4. Discussion

After analyzing the individual totals of confirmed COVID-19 cases and deaths in the states of Espírito Santo (ES) and Minas Gerais (MG), in the period from 2020 to 2022, it is clear that for the year 2021, the state of Minas Gerais recorded a higher number of cases, as well as deaths. For the state of Espírito Santo, the highest number of cases recorded during the pandemic period occurred in 2022; however, a lower number of deaths was already recorded.

When comparing the states, it is clear that, in proportion, ES had the highest incidence and mortality rates, while MG had the highest fatality rate.

Furthermore, in detail, there is a certain similarity with regard to the months that stood out due to the relative frequency in the distribution of cases and deaths for both states, with the vast majority being months at the beginning, middle, and end of the years. analyzed, such as January, February, June, July, November, and December. Another similar factor between states is the behavior of the increase in the number of deaths occurring in the current month or subsequent to the increase in the number of cases.

Nevertheless, the estimates demonstrated a stationary trend for incidence, mortality, and lethality in Espírito Santo in the year 2022, while in the state of Minas Gerais, there is a decreasing trend for incidence and mortality and an increasing trend for lethality in the same period. The daily increase in lethality is 1.39% (95%CI 1.11: 1.66).

Regarding the increase in the number of cases and deaths in months with specific holidays, a study carried out by Ren and Hwang [20] corroborates this research, which shows that approximately six to eight days after a holiday, there was an increase of 1.30 to 1.41 times the incidence rate. This expected time is directly related to the period of manifestation of symptoms and/or the results of the exam performed. Another study, carried out by Hadianfar et al. [21] also agrees with the results found, where an approximate increase of up to 1872 new cases was observed after holidays (*p* < 0.001).

A study conducted in the United States revealed that the period significantly impacting the number of confirmed cases corresponds to holidays such as New Year’s Day, Martin Luther King Day, Good Friday, Independence Day, Memorial Day, Labor Day, Christmas, elections, vacations, among others [20]. In this regard, it aligns with the data found in our research, considering that these months also have similar holidays in Brazil, such as Christmas, New Year’s, elections, and vacation periods, leading to an increase in the number of people circulating, as well as group gatherings.

In this context, the results of the present study demonstrate an increase in incidence, mortality, and lethality in November and December 2022. During this period, a major event that may have favored this increase in cases and deaths was the election. There were two rounds of presidential elections, where for both the state of Espírito Santo and the state of Minas Gerais, approximately 80% of eligible electorates in each state turned out to vote [22].

Other factors may also be associated with the increase in the number of cases and, consequently, the number of deaths. For Wu et al. [23], information on demographic density, average family income, percentage of the population in poverty, education, etc., provides support for a better understanding of the disease’s behavior. In this aspect, the state of Espírito Santo has a demographic density 2.38 times greater than the state of Minas Gerais, with 83.20 inhabitants/km^2^ against 35.02 inhabitants/km^2^ [15,16].

Demographic density is important in influencing the impacts of an epidemic, considering its population groups and their vulnerabilities, assuming that the greater the population density, the greater the difficulty of distancing, given that distancing is related to human behavior and is challenging, contributing to reducing or increasing the damage caused by virus transmission [24].

In this sense, it may be a factor that justifies the state of Espírito Santo presenting higher incidence and mortality rates when compared to the other states. On the other hand, a study carried out by Sutton et al. [25] revealed that mortality from COVID-19 was higher in regions with lower population density, linking this to two factors: rural regions and a higher proportion of elderly people in these regions. It is noteworthy that these specificities end up being important, as they point out that these standards contribute information for the implementation of specific health policies for these areas and populations.

With regard to intervention measures, Wong and Li [24] report that implementations can occur according to population density, thus considering more stringent policies in high-density areas, compared to those with lower density, which would result in optional measures.

As measures, many countries have implemented different types of blockades, some as compulsory, others with penalties applied to non-compliance. Among the measures, blockades in transport areas were adopted, such as stations, airports, and highways, in order to reduce population movement. Thus, Tsuboi, Fujiwara, and Itoh [26] state that blockades, information, and changes in the population’s behavior to the detriment of the pandemic contribute to reducing the risk of infection.

However, in order to effectively control the spread of the virus and consequently the disease, early implementation of measures is necessary. Among the measures, social distancing is influential in reducing daily cases of COVID-19, and for every day of delay in its implementation, there is a 2.41-day delay in containing the pandemic [21,27]. Corroborating this idea, Remuzzi and Remuzzi [28] argue that the exponential increase in mortality in the population may be linked to the late implementation of control measures.

A study carried out in different countries such as Spain, China, the United States, Japan, and Sweden, demonstrated that in 2021, compared to 2020, people reacted with less sensitivity to the suggested measures, arguing that in the first year of the pandemic, the measures were more effective [26].

When evaluating the results of our research, a discrepancy was noted compared to the previously mentioned study, as they advocate for greater adherence to the measures adopted, which contributed to the decrease in rates. However, in the states of Espírito Santo and Minas Gerais, there was an increase in the number of cases and deaths during the first year of the pandemic.

A study conducted in Curitiba, Brazil, spanning from 2020 to mid-2022, resulted in finding that in the year 2022, the incidence of COVID-19 was twice as high compared to previous years. However, lethality was already declining, being the lowest recorded during the pandemic period for this city [29].

Furthermore, with the emergence of new variants, especially Omicron, it was noted that in previously infected or vaccinated people in the United States, cases were classified as mild, which made it possible to reopen gathering spaces [30]. However, it appears that although there was a risk of an increase in the number of cases, the scenario did not increase lethality [31]. In view of this, Adamoski et al. [29] reinforce that, although it is milder, the increase in number can lead to an overload of the health service.

Also, the behavior of COVID-19 demonstrates a combination of several factors that influence its epidemiological dynamics, and the lingering effects of COVID-19 will continue to impact our communities, requiring sustained initiatives and interventions to provide comprehensive care to the population and promote overall well-being [32,33,34,35].

In this aspect, the process of understanding the course of the disease, as well as its trend throughout the pandemic period in the states of Espírito Santo and Minas Gerais, is understood as important, considering that the robustness of information is capable of bringing greater understanding about the disease, as well as on the interventions implemented over the years studied.

Such information, in addition to contributing to the present time, assuming that there are still records of new cases of COVID-19 in both states and that specific measures continue to be adopted, can support the process of evaluating the interventions carried out, as well as, new measures to be implemented in future epidemics, especially actions to promote, prevent, monitor and control the disease.

Given the stationary trend in most rates in 2022, it is suggested that the measures were important, from their first implementations to their revocations and/or changes, for maintaining a controlled state in a pandemic period, which reinforces the need for new studies to further understand the profile of the disease, thus ensuring that there are specific measures and better control of COVID-19.

The present study stands out for bringing a comparative analysis of epidemiological outcomes between two states in the period from 2020 to 2022; however, it presents aspects of limitations regarding the information analyzed, considering that the process of updating information in municipalities, states, and at the federal level it is dynamic and complex, where data undergoes changes every day. Furthermore, it is worth noting that this database may reflect numbers lower than the actual figures, given the possibility of underreporting in the number of COVID-19 cases and deaths.

## 5. Conclusions

There were 15,400 deaths in the state of Espírito Santo and 64,498 deaths in the state of Minas Gerais confirmed by COVID-19 in the period from January 2020 to December 2022.

When comparing the rates studied during this period, it was possible to observe that the state of Espírito Santo had a higher incidence of COVID-19, as well as higher mortality, when compared to the state of Minas Gerais. In turn, with regard to lethality, Minas Gerais stood out throughout the period of analysis, presenting the highest rate. Although divergent, both states had higher records in similar months, with specific months of greater festivities.

Furthermore, it was observed that both states showed equal trends for mortality, lethality, and incidence in the years 2020 and 2021. For the year 2022, Minas Gerais presents decreasing mortality and incidence and increasing lethality, while the state of Espírito Santo presents a stationary trend for all variables.

## Figures and Tables

**Table 1:** Monthly distribution of cases and deaths confirmed by COVID-19 in the state of Espírito Santo, Brazil, from January 2020 to December 2022.

Year	Month	Confirmed Cases		Confirmed Deaths	
		Frequency Absolute (*n*)	Frequency Relative (%)	Frequency Absolute (*n*)	Frequency Relative (%)
2020	January	0	0.00	0	0.00
	February	1	0.00	0	0.00
	March	151	0.01	0	0.00
	April	4101	0.31	116	0.75
	May	17,704	1.35	591	3.84
	June	35,880	2.73	1060	6.88
	July	37,305	2.84	904	5.87
	August	25,285	1.92	609	3.95
	September	19,108	1.45	397	2.58
	October	28,160	2.14	328	2.13
	November	46,414	3.53	505	3.28
	December	54,659	4.16	861	5.59
	Total	268,768	20.46	5371	34.88
2021	January	44,897	3.42	851	5.53
	February	30,527	2.32	568	3.69
	March	65,900	5.02	1274	8.27
	April	50,456	3.84	2112	13.71
	May	42,498	3.23	1273	8.27
	June	31,201	2.38	626	4.06
	July	21,211	1.61	377	2.45
	August	19,037	1.45	239	1.55
	September	24,322	1.85	266	1.73
	October	18,278	1.39	334	2.17
	November	13,116	1.00	215	1.40
	December	9852	0.75	122	0.79
	Total	371,295	28.26	8257	53.62
2022	January	255,645	19.46	298	1.94
	February	137,628	10.48	619	4.02
	March	12,073	0.92	171	1.11
	April	2394	0.18	29	0.19
	May	7262	0.55	21	0.14
	June	75,481	5.75	108	0.70
	July	77,148	5.87	231	1.50
	August	7938	0.60	85	0.55
	September	1372	0.10	19	0.12
	October	1202	0.09	6	0.04
	November	33,716	2.57	39	0.25
	December	61,788	4.70	146	0.95
	Total	673,647	51.28	1772	11.51
	Total	1,313,710	100.00	15,400	100.00

Source: Information extracted by the author from the Coronavirus Panel, (2023).

**Table 2:** Monthly distribution of cases and deaths confirmed by COVID-19 in the state of Minas Gerais, Brazil, from January 2020 to December 2022.

Year	Month	Confirmed Cases		Confirmed Deaths	
		Frequency Absolute (*n*)	Frequency relative (%)	Frequency Absolute (*n*)	Frequency Relative (%)
2020	January	139	0.00	1	0.00
	February	38	0.00	0	0.00
	March	1090	0.03	10	0.02
	April	3261	0.10	100	0.16
	May	13,596	0.40	305	0.47
	June	55,809	1.63	1035	1.60
	July	98,548	2.87	2360	3.66
	August	87,196	2.54	2365	3.67
	September	77,405	2.26	1941	3.01
	October	58,327	1.70	1499	2.32
	November	85,455	2.49	1160	1.80
	December	153,817	4.48	2560	3.97
	Total	634,681	18.50	13,336	20.68
2021	January	174,870	5.10	3730	5.78
	February	127,810	3.73	3329	5.16
	March	266,414	7.77	8483	13.15
	April	195,856	5.71	9262	14.36
	May	205,034	5.98	6220	9.64
	June	197,379	5.75	5100	7.91
	July	122,518	3.57	3243	5.03
	August	82,423	2.40	2006	3.11
	September	49,316	1.44	1143	1.77
	October	30,301	0.88	807	1.25
	November	17,368	0.51	388	0.60
	December	20,915	0.61	183	0.28
	Total	1,490,204	43.44	43,894	68.05
2022	January	568,480	16.57	1348	2.09
	February	302,803	8.83	2275	3.53
	March	49,652	1.45	739	1.15
	April	13,956	0.41	127	0.20
	May	63,177	1.84	157	0.24
	June	182,948	5.33	619	0.96
	July	116,177	3.39	829	1.29
	August	2792	0.08	345	0.53
	September	792	0.02	93	0.14
	October	381	0.01	45	0.07
	November	1253	0.04	111	0.17
	December	2803	0.08	580	0.90
	Total	1,305,214	38.05	7268	11.27
	Total	3,430,099	100.00	64,498	100.00

Source: Information extracted by the author from the Coronavirus Panel, (2023).

**Table 3:** Prais–Winsten regression estimates and daily change variation (DPC) of mortality, lethality, and incidence rates of COVID-19 in the states of Espírito Santo and Minas Gerais, Brazil, from January 2020 to December 2022.

Rate/Year	*β*	DPC (IC95%)	*p*	Trend
**Espírito Santo**				
**Mortality**				
2020	0.0023056	0.53 (0.16:0.90)	0.005 *	Growing
2021	−0.002948	−0.68 (−0.80:−0.55)	<0.001 *	Descending
2022	−0.0011957	−0.27 (−0.58:0.03)	0.076	Stationary
**Lethality**				
2020	−0.0019347	−0.44 (−0.57:−0.32)	<0.001 *	Descending
2021	−0.0008737	−0.20 (−0.29:−0.11)	<0.001 *	Descending
2022	−0.0000461	−0.01 (−0.39:0.37)	0.957	Stationary
**Incidence**				
2020	0.0084347	1.96 (1.26:2.67)	<0.001 *	Growing
2021	−0.0020619	−0.47 (−0.56:−0.38)	<0.001 *	Descending
2022	−0.0020398	−0.47 (−1.20:0.27)	0.209	Stationary
**Minas** **Gerais**				
**Mortality**				
2020	0.0063002	1.46 (1.03:1.90)	<0.001 *	Growing
2021	−0.0041913	−0.96 (−1.25:−0.67)	<0.001 *	Descending
2022	−0.002702	−0.62 (−1.07:−0.16)	0.008 *	Descending
**Lethality**				
2020	−0.0008331	−0.19 (−0.31:−0.07)	0.002 *	Descending
2021	−0.0008216	−0.19 (−0.29:−0.09)	<0.001 *	Descending
2022	0.0059761	1.39 (1.11:1.66)	<0.001 *	Growing
**Incidence**				
2020	0.0122493	2.86 (2.26:3.46)	<0.001 *	Growing
2021	−0.0034395	−0.79 (−0.93:−0.65)	<0.001 *	Descending
2022	−0.0078885	−1.80 (−2.35:−1.25)	<0.001 *	Descending

*β*—regression coefficient; *p*—*p*-value; DPC—daily percentage change; 95% CI—95% confidence interval. (*) Statistical difference detected by the Prais–Winsten regression test, *p* < 0.05. Source: prepared by the author (2023).

## Data Availability

Data supporting reported results can be found in the URL: https://docs.google.com/spreadsheets/d/1u7naOWd4O3YtDzP8j89LykfXqJyBWXFn/edit?usp=drive_link&ouid=104493025969896953886&rtpof=true&sd=true (accessed on 26 May 2024).
